# Correlated Alterations in Serotonergic and Dopaminergic Modulations at the Hippocampal Mossy Fiber Synapse in Mice Lacking Dysbindin

**DOI:** 10.1371/journal.pone.0018113

**Published:** 2011-03-23

**Authors:** Katsunori Kobayashi, Satomi Umeda-Yano, Hidenaga Yamamori, Masatoshi Takeda, Hidenori Suzuki, Ryota Hashimoto

**Affiliations:** 1 Department of Pharmacology, Nippon Medical School, Tokyo, Japan; 2 Japan Science and Technology Agency, Core Research for Evolutional Science and Technology, Kawaguchi, Saitama, Japan; 3 Department of Molecular Neuropsychiatry, Osaka University Graduate School of Medicine, Suita, Osaka, Japan; 4 Department of Psychiatry, Osaka University Graduate School of Medicine, Suita, Osaka, Japan; 5 Molecular Research Center for Children's Mental Development, United Graduate School of Child Development, Osaka University, Kanazawa University and Hamamatsu University School of Medicine, Suita, Osaka, Japan; Tokyo Institute of Psychiatry, Japan

## Abstract

Dysbindin-1 (dystrobrevin-binding protein 1, DTNBP1) is one of the promising schizophrenia susceptibility genes. Dysbindin protein is abundantly expressed in synaptic regions of the hippocampus, including the terminal field of the mossy fibers, and this hippocampal expression of dysbindin is strongly reduced in patients with schizophrenia. In the present study, we examined the functional role of dysbindin in hippocampal mossy fiber-CA3 synaptic transmission and its modulation using the sandy mouse, a spontaneous mutant with deletion in the dysbindin gene. Electrophysiological recordings were made in hippocampal slices prepared from adult male sandy mice and their wild-type littermates. Basic properties of the mossy fiber synaptic transmission in the mutant mice were generally normal except for slightly reduced frequency facilitation. Serotonin and dopamine, two major neuromodulators implicated in the pathophysiology of schizophrenia, can potentiate mossy fiber synaptic transmission probably via an increase in cAMP levels. Synaptic potentiation induced by serotonin and dopamine was very variable in magnitude in the mutant mice, with some mice showing prominent enhancement as compared with the wild-type mice. In addition, the magnitude of potentiation induced by these monoamines significantly correlated with each other in the mutant mice, indicating that a subpopulation of sandy mice has marked hypersensitivity to both serotonin and dopamine. While direct activation of the cAMP cascade by forskolin induced robust synaptic potentiation in both wild-type and mutant mice, this forskolin-induced potentaition correlated in magnitude with the serotonin-induced potentiation only in the mutant mice, suggesting a possible change in coupling of receptor activation to downstream signaling. These results suggest that the dysbindin deficiency could be an essential genetic factor that causes synaptic hypersensitivity to dopamine and serotonin. The altered monoaminergic modulation at the mossy fiber synapse could be a candidate pathophysiological basis for impairment of hippocampus-dependent brain functions in schizophrenia.

## Introduction

The dentate gyrus of the hippocampus has been implicated in the neuropathology of schizophrenia [1], [2]. The synapse formed by the mossy fiber, the axon of the dentate granule cell, is regulated by dopamine [3] and serotonin [4], [5], the neurotransmitters that have been implicated in the pathophysiolgy of schizophrenia [6], and has been suggested to be a potential target for pharmacological treatments of psychiatric disorders [2]. Since the mossy fiber plays an instructive role in the induction of associative synaptic plasticity at the recurrent excitatory synapses in its target CA3 region [7], [8], aberrant signal transmission and/or neuromodulation at the mossy fiber synapse in pathological conditions would impair proper functioning of the hippocampal neuronal circuit. An immunohistochemical study of the postmortem human brain has demonstrated a high protein expression level of dysbindin-1, one of the promising schizophrenia susceptibility genes [9], in both somatic and synaptic layers in the hippocampus [10]. In patients with schizophrenia, the hippocampal expression of dysbindin is greatly reduced, especially in the dentate gyrus and CA3 region [10], [11]. Dysbindin is a coiled-coil-containing protein, originally identified as a dystrobrevin binding protein [12] and later shown to be a component of the biogenesis of lysosome-related organelles complex 1, which regulates trafficking to lysosome-related organelles [13]. Although the physiological function of the dysbindin protein in the brain is not fully understood, it has been implicated in regulation of transmitter release [14]–[16], surface expression of the glutamate NMDA receptor [17] and the dopamine D_2_ receptor [18], [19], and synaptic plasticity [17]. Genetic variations in the dysbindin gene are associated with cognitive functions in humans [20], [21]. The reduction in the hippocampal expression of dysbindin might affect the dentate-to-CA3 signal transmission mediated by the mossy fiber, thereby possibly contributing to cognitive impairment and/or other symptoms of schizophrenia.

In the present study, in order to examine the functional role of dysbindin in the dentate granule cell-mossy fiber system, we used the sandy mouse, a spontaneous mutant in the DBA/2J strain with a 38,129-bp deletion including exons 6 and 7 in the dysbindin gene [13]. Sandy mice exhibit reduced locomotor activity in novel environments and increased anxiety-related behaviors in some conditions [22], [23]. Sandy mice also show behavioral abnormalities that are generally considered to be relevant to symptoms of schizophrenia, such as a deficit in social interaction [22], [24] and impairment of memory functions including working memory [16], [22]–[25]. We carried out electrophysiological recordings using hippocampal slices prepared from adult sandy mice and found marked abnormalities in monoaminergic modulations at the mossy fiber-CA3 synapse.

## Methods

### Ethics Statement

All procedures were approved by the Animal Care and Use Committee of Nippon Medical School (approval number 22-057).

### Electrophysiology

Sandy mice (dysbindin-1 mutant mice) were purchased from the Jackson Laboratory (Bar Harbor, Maine, USA). Mice were housed in the institutional standard condition (14∶10 light/dark cycle; lights on at 6:00 A.M. through 8:00 P.M.) at 23±1°C with food and water available ad libitum. Four- to six-month-old male sandy mice (−/−) and wild-type (+/+) littermates were used for experiments. Recordings and analyses were carried out essentially as described [3], [5]. Briefly, mice were decapitated under deep halothane anesthesia and both hippocampi were isolated. Transverse hippocampal slices (380 µm) were cut using a tissue slicer in ice-cold sucrose-containing saline composed of (in mM): sucrose 72, NaCl 80, KCl 2.5, NaH_2_PO_4_ 1.0, NaHCO_3_ 26.2, glucose 20, CaCl_2_ 0.5, MgCl_2_ 7 (equilibrated with 95% O_2_/5% CO_2_). Slices were maintained in a humidified interface holding chamber at room temperature (24–27°C) before recordings. Electrophysiological recordings were made in a submersion-type chamber maintained at 27.0–27.5°C and superfused at 2 ml/min with saline composed of (in mM): NaCl 125, KCl 2.5, NaH_2_PO_4_ 1.0, NaHCO_3_ 26.2, glucose 11, CaCl_2_ 2.5, MgCl_2_ 1.3 (equilibrated with 95% O_2_/5% CO_2_). The mossy fibers were activated by electrical stimulation delivered to the dentate granule cell layer, and field excitatory postsynaptic potentials (fEPSPs) arising from the mossy fiber synapses were recorded from the stratum lucidum of CA3. Single electrical stimulation was delivered at a frequency of 0.05 Hz unless otherwise specified, and the amplitude of fEPSPs was measured on analysis. A criterion used to identify the mossy fiber input was more than 85% block of fEPSP amplitude by an agonist of group II metabotropic glutamate receptors, (2S,2′R,3′R)-2-(2′,3′-dicarboxycyclopropyl)glycine (DCG-IV, 1 µM) (Tocris Bioscience, Bristol, UK). Whole-cell current-clamp recordings were made from the dentate granule cells with a pipette filled with a solution composed of (in mM): potassium gluconate 140, HEPES 20, NaCl 8, MgATP 2, Na_2_GTP 0.3, EGTA 0.05 (pH adjusted to 7.2 with KOH). The recording pipette was placed in the middle of the granule cell layer. Serotonin hydrochloride and forskolin were purchased from Sigma-Aldrich (St. Louis, MO, USA). Dopamine hydrochloride was from Wako Pure Chemical Industries, Ltd. (Osaka, Japan). All recordings were made using a Multiclamp 700B amplifier (Molecular Devices, Sunnyvale, CA, USA), filtered at 2–5 kHz and stored in a personal computer via an interface (digitized at 5–10 kHz). The number of data (n) represents the number of mice. Since some data from the mutant mice did not distribute normally, nonparametric two-tailed Mann-Whitney test was used to evaluate statistical significance, with the significance level *p*<0.05.

### RNA extraction, DNAse treatment and reverse transcriptase reaction

Tissues from the frontal cortex or hippocampus were homogenized in 4 mol/L guanidinium isothiocyanate (containing 25 nmol/L sodium citrate, pH 7.5, and 1% 2-mercaptoethanol), and total RNA was isolated by a standard phenol-chloroform extraction. The yield of total RNA determined by the absorbance at 260 nm and the quality of total RNA was also analyzed using agarose gel electrophoresis. Total RNA was treated with DNase for removal of contaminating genomic DNA using DNase Treatment & Removal Reagents (Ambion, Austin, TX, USA), according to the manufacturer's protocol. Total RNA (3.3 mg) treated with DNase was used in 50 ml of reverse transcriptase reaction to synthesize cDNA, by using a SuperScriptII First-Strand Synthesis System for RT-PCR (Invitrogen, Carlsbad, CA, USA), according to the manufacturer's protocol. Briefly, total RNA (3.3 mg) was denatured with 1 mM of dNTP and 6 ng/ml of random primers at 65°C for 5 min. After addition of RT buffer, dithiothreitol (10 mM in final concentration), RNAsin Plus RNase Inhibitor (40 units) and Super- ScriptII RT (200 units), the reaction mixture was incubated at 25°C for 10 min, at 42°C for 40 min, and at 70°C for 15 min. RNAse H (2 units) was added to the reaction mixture and then incubated at 37°C for 20 min.

### Real-time quantitative PCR

The TaqMan+ Endogenous Controls (Applied Biosystems, Foster City, CA, USA) were used for measurements of the house keeping gene GAPDH (Mm99999915_q1). TaqMan Gene Expression Assays (Applied Biosystems) were used for dopamine receptor D1A (Mm01353211_m1) and 5-hydroxytryptamine (serotonin) receptor 4 (Mm00434129_m1) genes. Both TaqMan assay kits included optimized concentrations of primers and probes to detect the target gene expression. The levels of mRNA expression of these genes were measured by a real-time quantitative RT-PCR using an ABI Prism 7900 sequence detection system with 384-well format (Applied Biosystems). Briefly, each 20 ml PCR reaction mixture contained 6 ml of cDNA, 0.5 ml of TaqMan assay kit and 10 ml of TaqMan Universal PCR Mastermix (Applied Biosystems). PCR cycling conditions were: 50°C for 2 min, 95°C for 10 min, 40 cycles of 95°C for 15 s and 60°C for 1 min. PCR data were obtained with the Sequence Detector Software (SDS version 2.1, Applied Biosystems) and quantified by a standard curve method. Standard curves were prepared using serial dilutions (1∶4) of pooled cDNA from total RNA derived from the whole brain of three mice.

## Results

**Figure 1:**
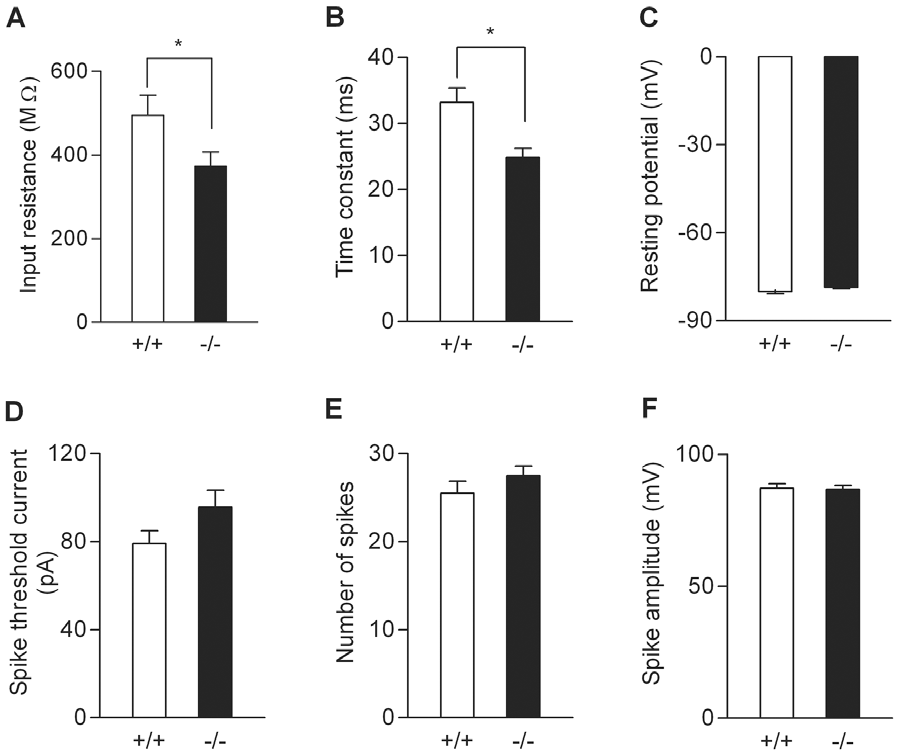
Passive and active somatic membrane properties of dentate granule cells. (A) Reduced input resistance in mutant mice (+/+: 494.8±48.4 MΩ, n = 12 cells; −/−: 373.6±33.4 MΩ, n = 14 cells; *p* = 0.0477).v (B) Shorter membrane time constant in mutant mice (+/+: 33.2±2.2 ms; −/−: 24.9±1.4 ms; *p* = 0.0109). (C–F) There was no significant difference in resting membrane potentials (C), the current intensity to evoke a single spike (D), the maximal number of spikes during sustained depolarization (E), or spike amplitude (F).

**Figure 2:**
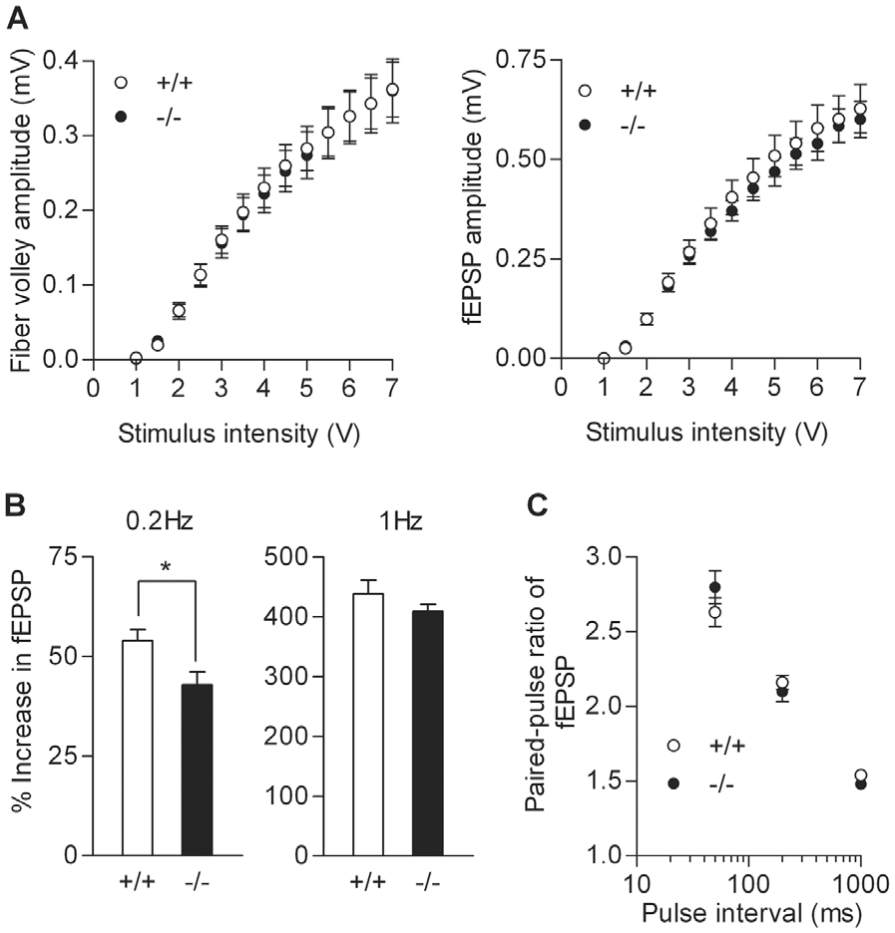
Basic properties of synaptic transmission at mossy fiber synapse. (A) Input-output relationship at the mossy fiber-CA3 synapse. The amplitude of the presynaptic fiber volley (left) and fEPSP (right) was plotted against the stimulus intensity (n = 15 slices each). (B) Frequency facilitation induced by repetitive stimulation was reduced in mutant mice at the stimulus frequency of 0.2 Hz (+/+: 54.0±2.8% increase, n = 8; −/−: 42.9±3.3% increase, n = 11; *p* = 0.039), but not at 1 Hz (+/+: n = 7; −/−: n = 10). (C) No significant difference in facilitation of fEPSP induced by paired stimulation (+/+: n = 8; −/−: n = 9).

**Figure 3:**
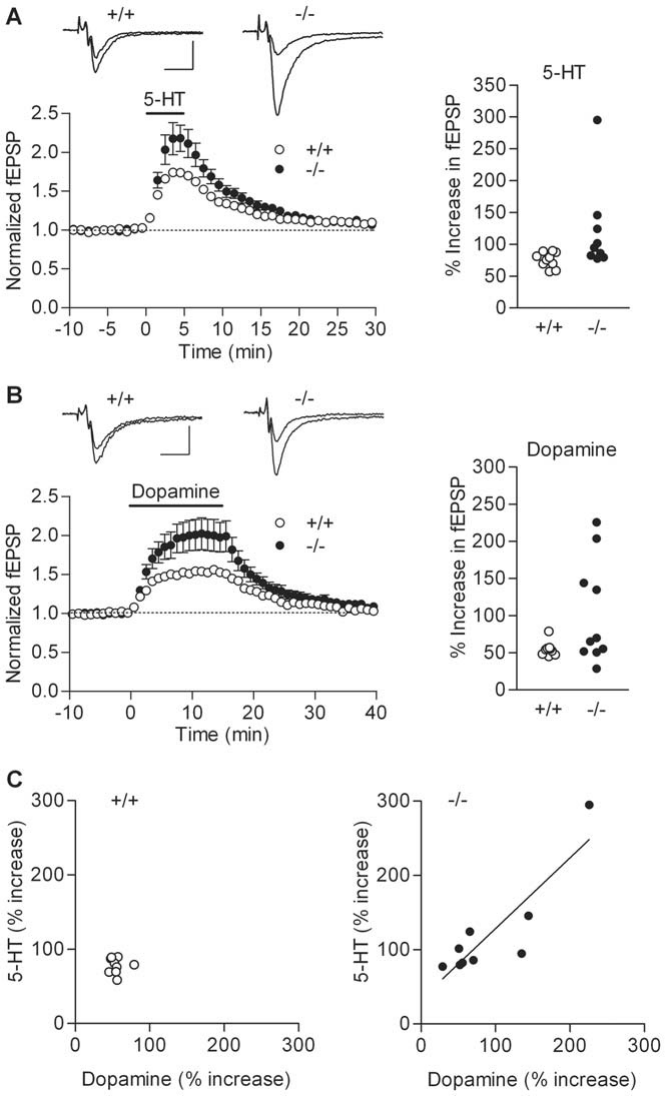
Altered monoaminergic synaptic modulations at mossy fiber synapse of sandy mice. (A) Left: Pooled data showing that serotonin-induced potentiation was enhanced in mutant mice (−/−:120.8±23.1% increase, n = 9) as compared with wild-type mice (+/+:75.9±3.8% increase, n = 10) (*p* = 0.0133). Serotonin (5-hydroxytriptamine, 5-HT) was applied at a concentration of 5 µM in bath for 5 min as indicated by the bar. Sample recordings are averages of 9 consecutive fEPSPs recorded during baseline and at the peak of potentiation. Scale bar: 10 ms, 0.2 mV. Right: Individual data showing the magnitude of serotonin-induced potentiation. Each symbol represents a single mouse. (B) Pooled (left) and individual (right) data showing potentiation of mossy fiber synaptic transmission by 10 µM dopamine (+/+: n = 9; −/−: n = 10). Some of mutant mice showed markedly enhanced synaptic potentiation, but there was no statistically significant difference (*p* = 0.1564). Scale bar: 10 ms, 0.2 mV. (C) Significant correlation between dopamine- and serotonin-induced synaptic potentiation in mutant mice (r^2^ = 0.7643, *p* = 0.002), but not in wild-type mice (r^2^ = 0.00098, *p* = 0.9362). Each symbol represents a single mouse.

We first examined basic electrophysiological properties of the dentate granule cell and the mossy fiber-CA3 synapse. Whole-cell recording from the granule cell soma revealed that the granule cells in the mutant mice had slightly reduced input resistance and membrane time constant (Figure 1A and 1B). However, there was no significant change in resting membrane potentials, the threshold current intensity to evoke a single action potential, the capability to generate multiple action potentials, or action potential amplitude (Figure 1C–1F). Thus, the somatic excitability of the granule cell is not appreciably changed in the mutant mice. Next, we analyzed fEPSPs arising from the mossy fiber-CA3 synapse. Synaptic responses were evoked by electrical stimulation in the dentate granule cell layer. There were no significant changes in the dependence of the presynaptic fiber volley and fEPSP amplitude on the stimulus intensity (Figure 2A), which is consistent with the intact somatic excitability of the mutant granule cells and also suggests that the basal transmission efficacy at the mossy fiber synapse is not altered. The mossy fiber synapse in adult mice is characterized by remarkable synaptic facilitation [2], [5], [26], a form of activity-dependent short-term synaptic plasticity. The mutant mice showed robust frequency facilitation and paired-pulse facilitation (Figure 2B and 2C). Although there was a small decrease in the magnitude of frequency facilitation at 0.2 Hz (Figure 2B), these results suggest that the mossy fiber synaptic transmission was also generally normal in the mutant mice. We have recently shown that the mossy fiber synaptic transmission is potentiated by dopamine [3] and serotonin [4], [5]. In the mutant mice, both serotonin and dopamine appeared to be more effective in inducing synaptic potentiation as compared with the wild-type mice (Figure 3A and 3B). In most cases, we examined effects of both serotonin and dopamine in the same mice, and noted that their effects were very variable and correlated with each other only in the mutant mice (Figure 3C). There was a statistically significant difference in the magnitude of serotonin-induced synaptic potentiation between the wild-type and mutant mice (Figure 3A). Although dopamine-induced synaptic potentiation was also clearly enhanced in some mutant mice, the difference did not reach the statistical significance probably due to the large variation (Figure 3B). Taken together, these results indicate that, while the basic properties of granule cells and mossy fiber synapses are largely preserved, a subpopulation of sandy mice has markedly enhanced sensitivity to both serotonin and dopamine at the mossy fiber synapse.

**Figure 4:**
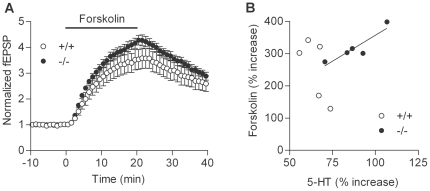
Synaptic potentiation induced by forskolin at mossy fiber synapse. (A) Pooled data showing potentiation of mossy fiber synaptic transmission by bath-applied forskolin (10 µM). There was no significant difference in the magnitude of potentiation between wild-type and mutant mice (*p* = 0.6905, n = 5 each). The effect of serotonin examined in the same mice was shown in (B) and was larger in mutant mice (*p* = 0.0159). (B) Significant correlation between serotonin- and forskolin-induced synaptic potentiation in mutant mice (r^2^ = 0.8177, *p* = 0.00351), but not in wild-type mice (r^2^ = 0.5067, *p* = 0.1775).

**Figure 5:**
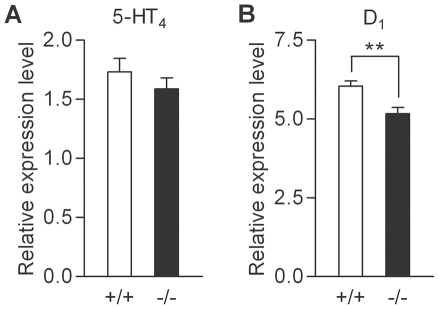
Reduced hippocampal expression of dopamine D1 receptor in sandy mice. (A) Expression levels of mRNAs for serotonin 5-HT_4_ receptor relative to those of GAPDH in the hippocampus (n = 10 each). (B) Relative expression levels of mRNAs for dopamine D_1_ receptor in the hippocampus were reduced in mutant mice as compared with wild-type mice (+/+: 6.04±0.17; −/−: 5.16±0.2, n = 10 each; *p* = 0.0068).

At the mossy fiber synapse, the potentiating effects of serotonin and dopamine are mediated by 5-HT_4_ and D_1_-like receptors, respectively [3]–[5]. These receptors are coupled to Gs-cAMP signaling cascades, and the mossy fiber synaptic potentiation induced by these monoamines has been shown to be mediated by cAMP [3], [4]. Next, we examined possible changes in the cAMP signaling cascade by using the adenylate cyclase activator forskolin. Bath-applied forskolin induced robust potentiation in both wild-type and mutant mice (Figure 4A). Although the magnitude of potentiation is slightly larger in the mutant mice, there was no statistically significant difference. However, we noticed that the magnitude of the forskolin-induced potentiation was significantly correlated with that of the serotonin-induced potentiation only in the mutant mice (Figure 4B). We also examined expression levels of mRNAs for 5-HT_4_ and D_1_ receptors in the hippocampus, and found a decrease in the expression level of the D_1_ receptor, but not of the 5-HT_4_ receptor (Figure 5).

## Discussion

The central serotonergic and dopaminergic systems are two major targets for the current pharmacological treatments for schizophrenia [6]. The present study demonstrated that the deletion mutation in the dysbindin gene can augment both dopaminergic and serotonergic synaptic modulations in the hippocampus, suggesting that the dysbindin deficiency could be an essential genetic factor that leads to alterations in these neuromodulatory systems in schizophrenia. However, the marked augmentation of the monoaminergic modulations was evident only in a subpopulation of the mutant mice. Dopamine potentiates the mossy fiber synaptic transmission by activating D_1_-like receptors [3], whose expression along the mossy fiber pathway is upregulated in mice heterozygous for the alpha-calcium/calmodulin-dependent protein kinase II [26]. The serotonergic modulation at the mossy fiber synapse can be strongly enhanced by chronic antidepressant treatment in adult mice [5]. It is generally believed that multiple genetic and environmental factors contribute to the pathogenesis of schizophrenia. If combined with such other factors, the dysbindin deficiency would more efficiently cause synaptic hypersensitivity to dopamine and serotonin.

In sandy mice, frequency facilitation was slightly reduced at the mossy fiber synapse. It is generally accepted that frequency facilitation is primarily mediated by an increase in the release of transmitters from presynaptic terminals [27]. The potentiating effects of both serotonin and dopamine at the mossy fiber synapse have been suggested to be mediated by presynaptic cAMP-dependent mechanisms [3], [4]. Therefore, it is likely that dysbindin is involved in regulation of presynaptic functioning at the mossy fiber synapse. While the effect of the adenylate cyclase activator foskolin was not significantly changed, the magnitude of potentiation induced by forskolin was significantly correlated with that by serotonin only in the mutant mice, raising the possibility that the receptor activation is more tightly coupled to the Gs-cAMP signaling in the mutant mice. This model may explain the correlated alteration of the serotonergic and dopaminergic modulation at the mossy fiber synapse in sandy mice. The PCR analysis revealed that the hippocampal expression of mRNAs for the D_1_ receptor, but not for the 5-HT_4_ receptor, is reduced in the mutant mice. This decrease in the receptor expression could cancel out possible changes in downstream signaling that can cause the enhancement of the synaptic modulation. Consistent with this idea, the enhancement of the dopamine-induced potentiation did not reach the statistical significance. Dopamine contents are reduced in the hippocampus of sandy mice [23]. The reduction in both dopamine levels and D_1_ receptor expression might have caused a compensatory modification of the downstream signaling. Cell surface expression of the dopamine D_2_ receptor is increased in cultured cortical neurons from dysbindin knockout mice [19]. Therefore, it is also possible that changes in the surface expression of the monoamine receptors on the mossy fiber terminals contributed to the altered synaptic modulation in sandy mice.

Sandy mice exhibit abnormalities in some forms of behaviors including working memory tasks [16], [22]. Patients with schizophrenia consistently show a deficit in working memory [28], and the dentate gyrus has been suggested to be essential for this cognitive function [29]. The augmented monoaminergic modulations at the mossy fiber synapse may cause exaggerated excitation of CA3 pyramidal cells upon activation of the dentate gyrus in some conditions. Such altered neuromodulations of the dentate-to-CA3 signal transmission may impair the performance of working memory in sandy mice and possibly in patients with schizophrenia.
